# High-resolution confocal imaging of wall ingrowth deposition in plant transfer cells: Semi-quantitative analysis of phloem parenchyma transfer cell development in leaf minor veins of Arabidopsis

**DOI:** 10.1186/s12870-015-0483-8

**Published:** 2015-04-23

**Authors:** Suong T T Nguyen, David W McCurdy

**Affiliations:** Centre for Plant Science, School of Environmental and Life Sciences, The University of Newcastle, Newcastle, NSW 2308 Australia

**Keywords:** Transfer cells, Cell wall ingrowths, Confocal imaging, Pseudo-Schiff base, Propidium iodide, Phloem parenchyma, Arabidopsis, Companion cells

## Abstract

**Background:**

Transfer cells (TCs) are *trans*-differentiated versions of existing cell types designed to facilitate enhanced membrane transport of nutrients at symplasmic/apoplasmic interfaces. This transport capacity is conferred by intricate wall ingrowths deposited secondarily on the inner face of the primary cell wall, hence promoting the potential trans-membrane flux of solutes and consequently assigning TCs as having key roles in plant growth and productivity. However, TCs are typically positioned deep within tissues and have been studied mostly by electron microscopy.

Recent advances in fluorophore labelling of plant cell walls using a modified pseudo-Schiff-propidium iodide (mPS-PI) staining procedure in combination with high-resolution confocal microscopy have allowed visualization of cellular details of individual tissue layers in whole mounts, hence enabling study of tissue and cellular architecture without the need for tissue sectioning. Here we apply a simplified version of the mPS-PI procedure for confocal imaging of cellulose-enriched wall ingrowths in vascular TCs at the whole tissue level.

**Results:**

The simplified mPS-PI staining procedure produced high-resolution three-dimensional images of individual cell types in vascular bundles and, importantly, wall ingrowths in phloem parenchyma (PP) TCs in minor veins of Arabidopsis leaves and companion cell TCs in pea. More efficient staining of tissues was obtained by replacing complex clearing procedures with a simple post-fixation bleaching step. We used this modified procedure to survey the presence of PP TCs in other tissues of Arabidopsis including cotyledons, cauline leaves and sepals. This high-resolution imaging enabled us to classify different stages of wall ingrowth development in Arabidopsis leaves, hence enabling semi-quantitative assessment of the extent of wall ingrowth deposition in PP TCs at the whole leaf level. Finally, we conducted a defoliation experiment as an example of using this approach to statistically analyze responses of PP TC development to leaf ablation.

**Conclusions:**

Use of a modified mPS-PI staining technique resulted in high-resolution confocal imaging of polarized wall ingrowth deposition in TCs. This technique can be used in place of conventional electron microscopy and opens new possibilities to study mechanisms determining polarized deposition of wall ingrowths and use reverse genetics to identify regulatory genes controlling TC *trans*-differentiation.

**Electronic supplementary material:**

The online version of this article (doi:10.1186/s12870-015-0483-8) contains supplementary material, which is available to authorized users.

## Background

Transfer cells (TCs) are important for plant development as they form at nutrient transport bottlenecks where an apoplasmic/symplasmic transport step is required for acquisition and/or delivery of nutrients [1]. TCs are anatomically specialized for this function as they develop extensive wall ingrowths which result in increased plasma membrane surface area which supports an increased density of nutrient transporters [1-3]. Seeds of many crop species develop TCs to facilitate seed filling [4], and TCs support both phloem loading and short and long distance transport via xylem/phloem exchange [5]. TCs develop by *trans*-differentiation of existing cell types in response to developmental or stress-induced signals [1], but despite the importance of TCs to plant development, little is known of the molecular processes responsible for their *trans*-differentiation. This situation is caused in part by TCs typically being located deep within tissues [6] and thus not readily accessible for experimental manipulation and study.

The *trans*-differentiation of TCs involves differential expression of hundreds of genes. The formation of nucellar projection and endosperm TCs in barley grains involves differential expression of at least 815 genes [7], while the development of epidermal TCs in *Vicia faba* cotyledons is predicted to involve up to 650 genes [8]. These and other observations have led to the proposition that wall ingrowth deposition in TCs involves hierarchical regulation of cascades of gene expression, presumably controlled by key transcription factors [9], a model based on the genetic regulation of secondary wall deposition in xylem tissue [10,11]. The identification of such factors putatively regulating wall ingrowth deposition in TCs is best undertaken in a genetic model such as *Arabidopsis thaliana* (Arabidopsis).

In Arabidopsis, phloem parenchyma (PP) TCs are known to form in minor veins of leaves and sepals where they are proposed to function in apoplasmic phloem loading [12-14]. Previous studies examining PP TCs in Arabidopsis have relied on transmission electron microscopy (TEM) to analyze these cells. Indeed, Amiard et al. [13] traced cell wall contours of PP TCs viewed by TEM to demonstrate a role for high light and jasmonic acid in signaling wall ingrowth development, and similar approaches were undertaken to demonstrate a relationship between photosynthetic capacity and PP TC development [15]. Analysis by electron microscopy, however, is time-consuming and clearly not compatible for high-throughput screening required to identify genetic factors controlling the *trans-*differentiation of TCs.

High-resolution imaging of cell walls by confocal microscopy has been achieved using a modified pseudo-Schiff base-propidium iodide (mPS-PI) staining procedure [16]. In this process, treatment of fixed plant tissue with periodic acid results in the formation of aldehyde groups in the carbohydrate moieties of cells walls. These aldehyde groups can then be reacted with various fluorescent pseudo-Schiff reagents, such as propidium iodide, resulting in strong covalent fluorophore labelling of cell walls [16]. The strong covalent labelling enables the tissue to be extensively cleared and mounted in high-refractive index mounting medium, giving strong and stable fluorescence labelling of cell walls and thus enabling extensive *z*-stack imaging of cellular organization in complex tissues [16,17]. Wall ingrowths of TCs are rich in cellulose and other polysaccharides such as pectins [18], a feature that may provide an opportunity to use the mPS-PI procedure to image wall ingrowth deposition in PP TCs.

Here we report the successful use of mPS-PI staining of Arabidopsis leaves to visualize wall ingrowth deposition in PP TCs in minor veins of leaves, cotyledons and sepals by confocal imaging. Wall ingrowths in these cells are discernable as highly localized thickenings of wall material deposited along the face of the PP TC adjacent to neighboring cells of the sieve element/companion cell (SE/CC) complex. Depending on tissue orientation, this deposition can often be seen as a central band running along each PP TC and superimposing an underlying SE or CC. We have used this procedure to also image light-dependent wall ingrowth deposition in CC TCs of pea minor veins [19,20], and have developed a scoring method based on the extent of wall ingrowth deposition for semi-quantitative analysis of TC development. Furthermore, introduction of a simple post-fixation bleaching step as an alternative to extensive clearing procedures in the original technique has simplified the processing steps to enable more efficient staining of tissue. Collectively, this procedure now provides the opportunity to investigate the cell biology of wall ingrowth deposition of PP TCs in Arabidopsis in a semi-quantitative manner without resorting to electron microscopy, and will also enable high-throughput phenotypic screening of TC development to identify key transcriptional regulators of this process.

## Results

### A modified pseudo-Schiff staining technique using propidium iodide to visualize wall ingrowths in TCs

**Figure 1:**
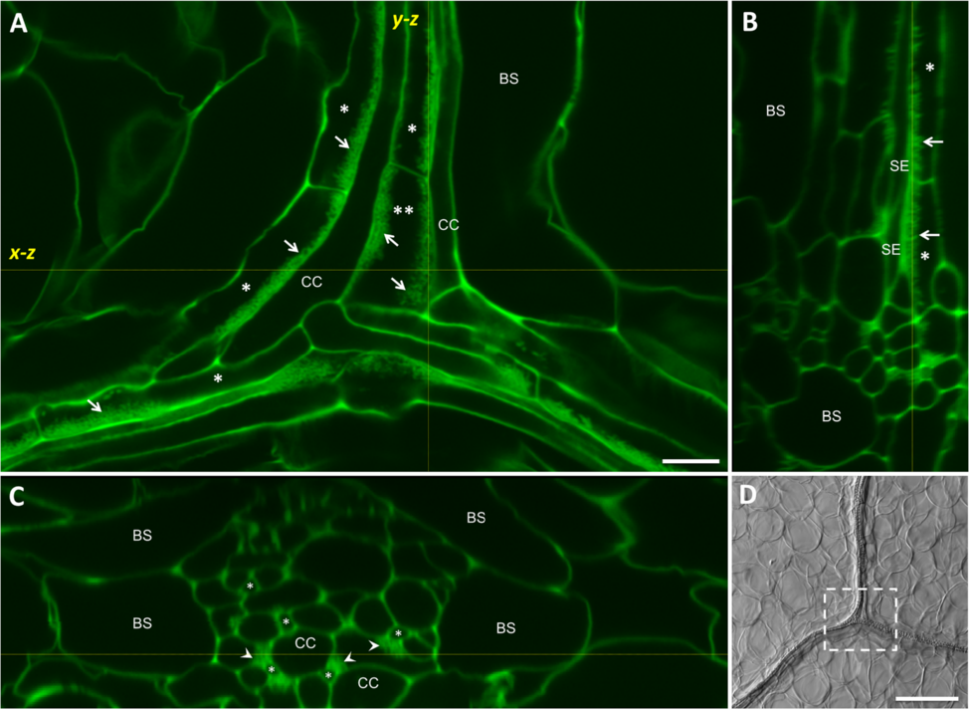
Confocal imaging of wall ingrowths in PP TCs of Arabidopsis leaf minor veins stained by the mPS-PI procedure. **A**. Single confocal section of a minor vein junction revealing polarized deposition of wall ingrowths (arrows) on the face of PP TCs (single asterisks) adjoining CCs. Polarized deposition of wall ingrowth material can be seen in other PP TCs, including the central PP TC (double asterisk) where ingrowth deposition is directed to opposite faces of the PP TC, each adjoining a different CC. The yellow dotted lines labelled *y-z* and *x-z* correspond to the projections shown in **B** and **C**, respectively. **B**. *y-z* projection of a *z*-stack of the image shown in **A** revealing finger-like projections (arrows) of wall ingrowth material extending from the face of two linearly-arranged PP TCs (asterisks) adjacent to neighboring SEs. **C**. *x-z* projection of a *z*-stack of the image shown in **A** revealing minor vein architecture in transverse section and the presence of highly-localized depositions of wall ingrowth material (arrowheads) adjacent to small SEs (asterisks) and larger CCs. **D**. Bright-field image of minor vein junction. The boxed area corresponds to the region shown in **A** and indicates the clarity of viewing vascular tissue when the abaxial epidermal layer is removed and the tissue is viewed from the abaxial surface of the leaf. BS, bundle sheath cell; CC, companion cell; SE, sieve element. Scale bar = 10 μm in **A**, **B** and **C**. Scale bar = 100 μm in **D**.

The development of high-contrast staining of cell walls in cleared plant tissue using a mPS-PI procedure has enabled improved confocal imaging throughout plant tissues generally [16] and leaf tissue in particular [17]. To develop a procedure for confocal imaging of wall ingrowths in PP TCs in Arabidopsis leaves we used the technique of Wuyts et al. [17], modified by first peeling away the abaxial epidermis of rosette leaves immediately prior to fixation. Removing the abaxial epidermal layer and most of the associated mesophyll tissue and viewing from the abaxial face of the leaf enabled clear viewing of vascular bundles (Figure 1D). Under these conditions, confocal imaging of mPS-PI-stained leaves clearly resolved bands of wall ingrowth material, seen as unevenly thickened and mottled staining, positioned along the face of PP TCs adjacent to cells of the SE/CC complex (Figure 1A). In these images PP TCs can be identified as relative thin, elongated cells sharing a common longitudinal wall with a larger bundle sheath cell and the opposite wall with neighboring cells of the SE/CC complex. The highly localized deposition of wall ingrowths in PP TCs is evidenced by their occurrence only along the wall shared with a cell of the SE/CC complex (Figure 1A,B). In Figure 1A, the PP TC labelled with a double asterisk shows localized ingrowth deposition on the two faces of the cell neighboring different CCs, indicating that the localizing signal most likely emanates from cells of the SE/CC complex. A longitudinal *y-z* projection of a *z*-stack through the vascular bundle shown in Figure 1A resolved discrete finger-like projections of wall ingrowth material along the face of PP TCs neighboring two SEs (Figure 1B). This image is highly reminiscent of TEM views of finger-like wall ingrowth projections in these cells (see Figure six of [21]), thus supporting the conclusion that the structures being imaged are indeed wall ingrowths in PP TCs. An *x-z* projection of the same *z*-stack showing the vascular bundle in transverse section, clearly resolved localized patches of wall ingrowth material deposited in PP TCs adjacent mostly to the smaller SEs but also to CCs (Figure 1C). A survey of PP TCs revealed that in most discernable instances, wall ingrowth deposition in a PP TC was initiated immediately opposite a SE, but consolidation of this deposition spreads to areas of the cell wall opposite CCs (data not shown). This observation supports the suggestion that the source of signals such as reactive oxygen species likely to drive wall ingrowth deposition in PP TCs is derived from SEs [5].

**Figure 2:**
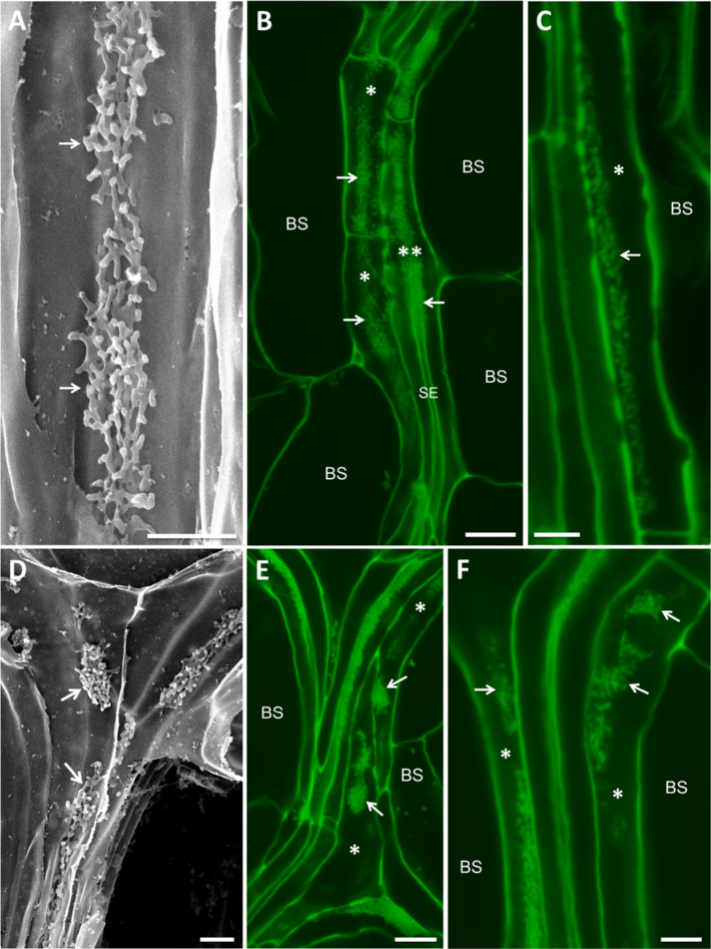
Comparison of wall ingrowths in PP TCs of Arabidopsis leaf minor veins by confocal imaging and SEM. **A**, **D**. SEM views of fresh leaf material torn paradermally then subjected to bleach extraction and viewed by SEM. **B**, **C**, **E**, **F**. Confocal imaging of minor veins from leaf material stained by the mPS-PI procedure. **A**. SEM image of a PP TC showing a central band of reticulate wall ingrowth material (arrows). **B**. Highly localized deposition of wall ingrowth material seen as a central band (arrows) running along the length of each PP TC (asterisks). The focal plane of the image passes from the PP TC on the right (double asterisk) into the underlying SE, indicating how the band of wall ingrowth material superimposes the underlying SE. **C**. Confocal image at higher magnification showing substructure (arrow) of the wall ingrowth material in a PP TC (asterisk). In this image the SE to the left of this PP TC is obscured. **D**. SEM view of a minor vein junction of two PP TCs showing examples of isolated patches of wall ingrowth deposition (arrows). **E**. Confocal image of minor vein junction showing discrete patches of wall ingrowth deposition (arrows). **F**. Higher magnification confocal image showing patches of wall ingrowth deposition (arrows) in two PP TCs (asterisks). BS, bundle sheath cell; CC, companion cell; SE, sieve element. Scale bars = 2 μm in **A** and **D**. Scale bars = 10 μm in **B** and **E**. Scale bars = 5 μm in **C** and **F**. The image in **A** is reproduced in part from Edwards et al. [22].

When rosette leaves are torn paradermally and viewed by scanning electron microscopy (SEM), wall ingrowth deposition can often be seen as a central band of reticulate ingrowth material running along the length of a given PP TC (Figure 2A; see [22]), or as discrete clumps of tangled, finger-like projections (Figure 2D). These features are also seen by confocal imaging of mPS-PI-stained leaf material, namely central bands of wall ingrowth material running along PP TCs (arrows, Figure 2B) and isolated clumps of wall ingrowths (arrows, Figure 2E,F). The central bands of ingrowth material reflect their highly localized deposition immediately adjacent to neighboring cells of the SE/CC complex (see [12,13]). This spatial relationship is clearly seen in Figure 2B where the focal plane in the middle of the image passes from a PP TC (double asterisk, Figure 2B) to an underlying SE, revealing how the band of wall ingrowth material in the PP TC superimposes the underlying SE (Figure 2B). This feature is particularly evident when viewed as a *z*-stack movie through these cells (Additional file 1: Movie S1). At higher magnification, confocal imaging clearly resolved the intertwined, finger-like projections of wall ingrowth material (arrow, Figure 2C), a feature that is readily evident when viewed by SEM (Figure 2A).

We surveyed different vein orders in mature leaves for the presence of PP TCs. Consistent with previous studies identifying phloem involved in assimilate loading in mature leaves [23], we observed typically substantial levels of wall ingrowth deposition in virtually all veins except the midrib and most of the secondary veins (data not shown). The exception to this observation was minor levels of wall ingrowth deposition seen in small terminating regions of secondary veins (Additional file 2: Movie S2). These observations are consistent with the conclusion that wall ingrowth deposition in PP TCs correlates with phloem loading capacity of minor veins [23], which in turn correlates inversely with vein size, as suggested by Haritatos et al. [12].

### Imaging wall ingrowth deposition in CC TCs in leaf minor veins of pea

**Figure 3:**
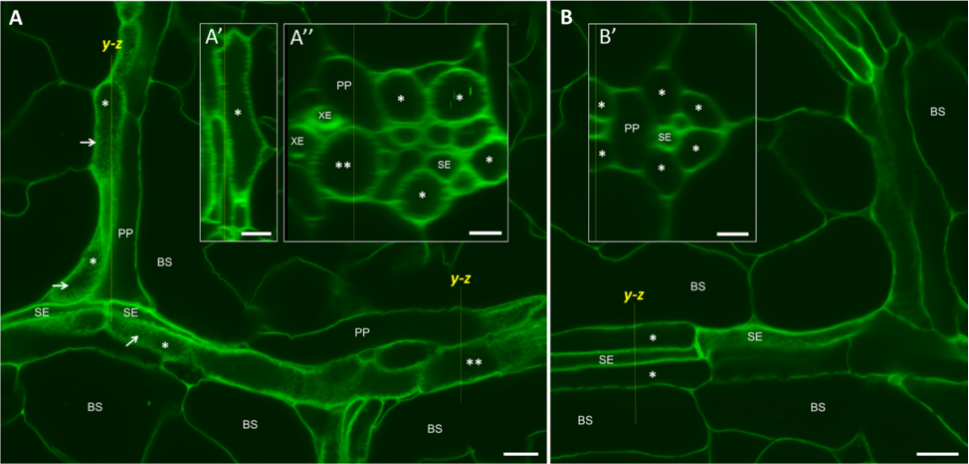
Confocal imaging of wall ingrowth deposition in CC TCs of leaf minor veins in pea. **A**. Minor veins from second true leaf of 13 day-old full light-grown seedlings showing mottled wall ingrowth labelling across the full face of CC TCs (asterisks). No wall ingrowth deposition is seen in PP cells. A *y-z* projection of a *z*-stack through the vertical minor vein is shown in inset A’. Wall ingrowth deposition detected as fuzzy labelling can be seen along all longitudinal walls of the CC TC (asterisk). A *y-z* projection through the horizontal minor vein at the bottom right of **A** is shown in inset A”. Here, fuzzy labelling indicating wall ingrowth deposition is seen around all faces of the CC TCs seen in transverse view. The double asterisk in **A** and A” indicates a large CC TC. **B**. Minor veins from second true leaf of 9 day-old seedling subjected to 4 days of darkness. No wall ingrowth deposition is seen in CC TCs (asterisks) seen in **B** or when the minor vein is seen in transverse view as a *y-z* projection through this minor vein (inset B’). BS, bundle sheath cell; PP, phloem parenchyma; SE, sieve element; XE, xylem element. Scale bars = 10 μm in **A**, A’ and **B**. Scale bars = 5 μm in A” and B’.

To test the general applicability of this method for imaging wall ingrowths in other species, we used the mPS-PI procedure of Wuyts et al. [17] to stain CC TCs in leaf minor veins of pea, where reticulate wall ingrowth deposition occurs on all faces of these cells [19,20,24]. Confocal imaging of minor veins showed mottled labelling across the full face of CC TCs (arrows, Figure 3A). In contrast, wall ingrowth deposition was not detected in PP cells neighboring cells of the bundle sheath (Figure 3A). A *y-z* projection of a *z*-stack passing longitudinally through the vertically-orientated minor vein in Figure 3A revealed the presence of reticulate wall ingrowths along the longitudinal walls of a CC TC (inset A’, Figure 3A), and similarly, a *y-z* projection passing transversely through the horizontal minor vein in Figure 3A revealed wall ingrowth deposition across all faces of the large, mostly circular CC TCs (inset A”, Figure 3A), consistent with TEM images of these cells [20,24]. To verify that these structures were indeed wall ingrowths, leaves were stained from light-grown plants subjected to 4 days of dark treatment, conditions known to cause reduced wall ingrowth deposition [19,20]. Accordingly, reticulate wall ingrowth deposition in CC TCs was also greatly reduced, as shown by the *y-z* projection of a *z*-stack passing transversely through a vascular bundle (inset B’, Figure 3B). This result confirms that the CC TCs shown in Figure 3A contain reticulate wall ingrowths, and that these ingrowths can be detected by confocal imaging of mPS-PI-stained pea leaves.

### A simplified extraction procedure for mPS-PI staining of PP TCs using sodium hypochlorite

The collective analyses described above established that the mPS-PI staining procedure adapted from Wuyts et al. [17] can be used to image wall ingrowth deposition in TCs involved in phloem loading in both Arabidopsis and pea. The Wuyts et al. procedure is lengthy, however, involving several extractions in organic solvents and clearing in SDS/NaOH, followed by overnight treatment with amylase and pullulanase prior to mPS-PI staining (see Methods). To circumvent these lengthy procedures, we tested bleaching of fixed and ethanol-washed leaf tissue in sodium hypochlorite to clear cellular content for subsequent mPS-PI-staining of cell walls. Sodium hypochlorite was used by Sugimoto et al. [25] to extract cellular content from root tissue prior to viewing cellulose microfibrils by field emission SEM, and was used by Edwards et al. [22] to clear leaf tissue for fluorescence imaging of wall ingrowth deposition in PP TCs using Calcofluor White. In this current study, the use of bleach to clear tissue resulted in equivalent, if indeed somewhat improved, imaging of wall ingrowths in PP TCs (data not shown) compared to the procedure of Wuyts et al. The bleach method provided consistently good extraction of cellular content, with the minor exception of cotyledons (see below), and often yielded well defined cellular morphology as revealed by a *z-*series scan of vascular tissue (Additional file 3: Movie S3). Given this outcome, we subsequently adopted the bleaching of fixed and ethanol-washed tissue as our standard method for confocal imaging of mPS-PI-stained tissue.

### Wall ingrowth deposition in PP TCs from different tissues of Arabidopsis

**Figure 4:**
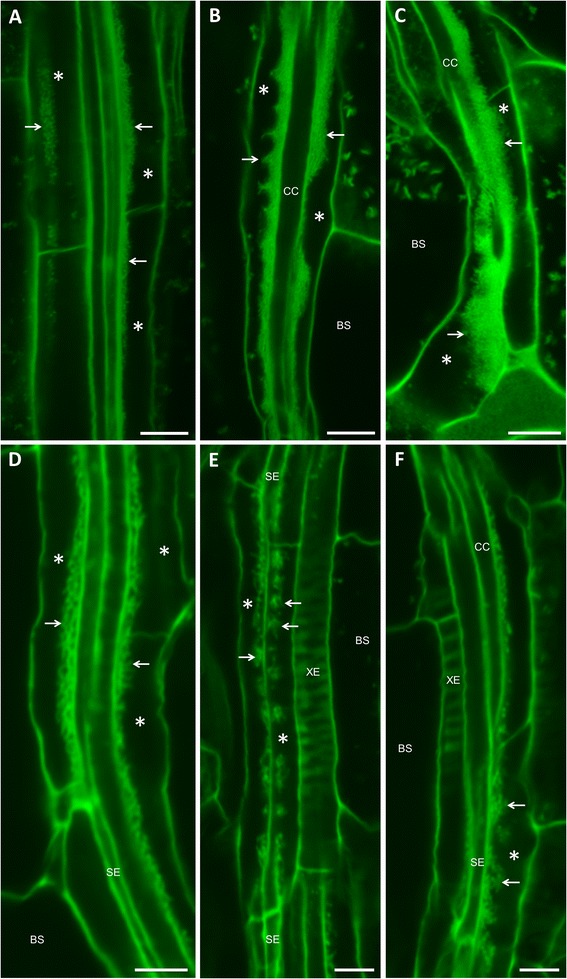
Confocal imaging of wall ingrowth deposition in PP TCs in cotyledons, cauline leaves and sepal. **A**. Minor vein from the base of an 18 day-old cotyledon showing extensive wall ingrowth deposition (arrows) in PP TCs (asterisks). **B**. Minor vein from the mid-region of an 18 day-old cotyledon showing highly sculptured and extensive wall ingrowth deposition (arrows) in PP TCs (asterisks). **C**. Minor vein from the tip an 18 day-old cotyledon showing massive levels of wall ingrowth deposition (arrows) that occupy a considerable volume of each PP TC (asterisks). The fragments of fluorescent labelling seen in bundle sheath cells in both **B** and **C** correspond to remnant starch grains not completely extracted by the bleach treatment. **D**. Wall ingrowth deposition (arrows) in PP TCs (asterisks) in a fully expanded cauline leaf. **E**. Wall ingrowths in a minor vein of sepal, showing numerous localized patches of wall deposition (arrows) along each PP TC (asterisk). **F**. Wall ingrowths in a minor vein of sepal showing apparent consolidation or merging of localized patches of wall ingrowth material (arrows) in a PP TC (asterisk). BS, bundle sheath cell; CC, companion cell; XE, xylem element. Scale bars = 10 μm in **A**, **B** and **C**. Scale bars = 5 μm in **D**, **E** and **F**.

We used our modified mPS-PI-staining procedure to survey other tissues in Arabidopsis for the presence of PP TCs. In cotyledons, as typically seen in rosette leaves and other tissues (see below), the morphology of wall ingrowths can vary depending on age of the tissue. For example, early-stage wall ingrowth development in cotyledons of 7 day-old seedlings appears identical to early-stage wall ingrowth deposition in immature leaves of 14 day-old seedlings (data not shown). In cotyledons from 18 day-old seedlings, however, extensive deposition of wall ingrowths is seen along the face of PP TCs adjacent to cell*s* of the SE/CC complex (Figure 4A-C). The morphology of wall ingrowth deposition in cotyledons from such plants was surprisingly varied, ranging from uniform deposition similar to that seen in rosette leaves (Figure 4A, Additional file 4: Figure S1A), to sharply pointed peaks of wall ingrowth material (Figure 4B), or very substantial deposition, albeit irregularly distributed along the length of a given PP TC and occupying a considerable volume of the cell (Figure 4B,C). This feature is similar to the manner in which dense fenestrated networks of ingrowth material protrude extensively into the outer periclinal cytoplasmic volume of abaxial epidermal TCs in *V. faba* cotyledons [26]. The images shown in Figure 4A-C are of PP TCs in vascular bundles located at the base, middle and tip regions of cotyledons, respectively, reflecting a basipetal gradient of wall ingrowth deposition which correlates with phloem loading capacity in cotyledons [27]. Variations in wall ingrowth development are also apparent in nearby veins as seen in Additional file 4: Figure S1A. The PP TC marked with an asterisk in Figure S1A and A’ developed very extensive and dense wall ingrowths, while in a nearby PP TC (double asterisk, Additional file 4: Figure S1A) wall ingrowth deposition was less developed, hence typical finger-like projections can be detected in a longitudinal view (double asterisk, Additional file 4: Figure S1A”) reconstructed from the *z*-stack image shown in S1A.

**Figure 5:**
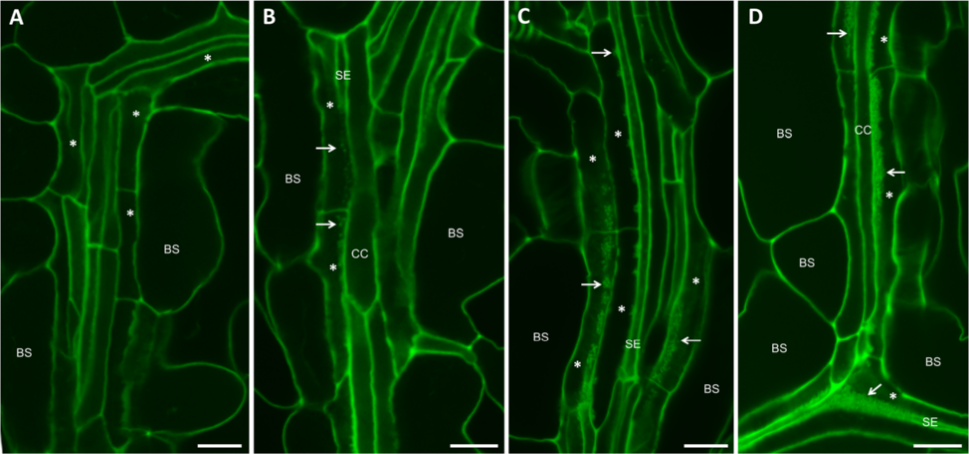
Classification system for the extent of wall ingrowth deposition in PP TCs in minor veins of Arabidopsis leaves. **A**. Class I - no wall ingrowths visible in PP cells (asterisks), which are identified by their sharing a common wall with a large bundle sheath cell. **B**. Class II - evidence of discrete, punctate-like wall ingrowth deposition seen as discrete fluorescent dots (arrows) distributed along the face of PP TCs adjacent to either a CC or SE. Not all PP cells in a given field of view contain wall ingrowths. **C**. Class III - substantial levels of reticulate wall ingrowth deposition is seen as clusters or continuous stretches of fluorescent labelling (arrows) on the face of PP TCs (asterisks) neighboring cells of the SE/CC complex. This level of labelling is seen in most PP cells in a given field of view, but can be somewhat variable. **D**. Class IV - extensive wall ingrowth deposition seen as thick bands of fluorescence labelling (arrows) seen in essentially all PP TCs in a given field of view. BS, bundle sheath cell; CC, companion cell; SE, sieve element. Scale bars = 10 μm in **A**, **B**, **C** and **D**.

Wall ingrowth deposition in PP TCs was also detected in cauline leaves (Figure 4D, Additional file 4: Figure S1B) and sepals (Figure 4E,F, Additional file 4: Figure S1C). In cauline leaves deposition of ingrowth material was typically abundant, especially in veins in the tip region of the leaf (Figure 4D). In sepal tissue, wall ingrowths were often seen as discrete clusters of wall material positioned along the length of a PP TC (Figure 4E). These discrete clusters appeared in places to merge with neighboring clusters to form localized clumps of ingrowth material along a given PP TC (Figure 4F). These features were also seen in PP TCs in other tissues (e.g., Figure 5C), but were more common in sepals. Xylem elements were often detected adjacent to PP TCs in sepals (Figure 4E,F, Additional file 4: Figure S1C) due to the simple structure of vascular bundles in this tissue (see [14]).

### Semi-quantitative assessment of wall ingrowth deposition in PP TCs

The clarity of confocal imaging by the bleach-modified mPS-PI procedure enabled semi-quantitative assessment of both the extent of *trans-*differentiation of PP TCs and the abundance of wall ingrowth deposition in a given cell. To facilitate this process, we developed a scoring procedure ranging across four categories of wall ingrowth deposition as defined by our observations. Class I represents PP cells with no detectable wall ingrowths (Figure 5A, Additional file 5: Figure S2A, B). These cells, defined by their elongated, rectangular shape and connection to a neighboring bundle sheath cell, were devoid of detectable wall ingrowths as evidenced by the thin, regular outline of their stained cell walls. In Class II, wall ingrowths were detected in early stages of development as evidenced by limited regions of patchy, mottled staining along the wall of the PP TC opposite that of a bundle sheath cell (Figure 5B, Additional file 5: Figure S2F), or visualized as discrete dots of fluorescence in face view (Additional file 5: Figure S2C, D, E). For this class, not all PP cells in a given region of vein showed evidence of wall ingrowth deposition. In Class III, wall ingrowth deposition was more obvious as wider regions of mottled fluorescence and this level of deposition was commonly detected in most but not necessarily all PP cells in a given field of view (Figure 5C, Additional file 5: Figure S2G, H, I, J). In Class IV, wall ingrowths in PP TCs were very abundant and seen as continuous thick bands of mottled fluorescence present in essentially all PP TCs in the field of view (Figure 5D, Additional file 5: Figure S2K, L).

**Figure 6:**
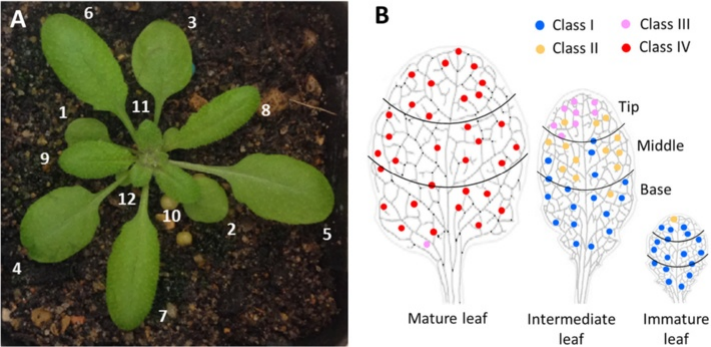
Survey of wall ingrowth deposition in PP TCs in minor veins across leaf development in Arabidopsis. **A**. Leaf numbering of 3.5 week-old rosette leaf. **B**. Representation of wall ingrowth deposition in immature, intermediate and mature leaves as represented by color-coding of the four classes of wall ingrowth deposition as described in Results. The result shown here is representative of three replicate leaves for each stage of development. Immature leaves contain little if any wall ingrowth deposition in PP cells. Intermediate leaves show a basipetal gradient from typically Class III at the tip to Class I at the leaf base. Mature leaves contain Class IV wall ingrowth deposition in PP TCs of minor veins throughout the entire leaf, with the exception of a few examples of Class III at the very base of the leaf. Skeletonized images of leaves shown in Figure 6B were adapted from Alonso-Peral et al. [34].

Using this classification system, we qualitatively surveyed the abundance of wall ingrowth deposition in PP TCs in terminating minor veins across leaf development, selecting leaf 11, 8 and 5 as representative of immature, intermediate and mature leaves, respectively (Figure 6A). A representation of this survey is shown in Figure 6B. Immature sink leaves contain predominantly Class I PP cells with no wall ingrowths, while intermediate leaves are characterized by a basipetal gradient with Class III PP TCs in minor veins at the tip of the leaf and predominantly Class I PP cells at the leaf base. In contrast, mature source leaves are dominated by Class IV PP TCs with highly abundant wall ingrowths in minor veins across virtually the entire leaf (Figure 6B). The distribution of PP TCs seen in this analysis is consistent with the known sink-source transition that occurs in maturing leaves [28], as well as the development of apoplasmic loading that occurs in a basipetal gradient within a dicot leaf [23,27]. The value of mPS-PI staining in this case, however, is that it provides a rapid means to assess the development of PP TCs across an entire leaf in response to different biotic or abiotic signals and within different genetic backgrounds, with high spatial resolution without relying on time-consuming procedures such as TEM.

### Manipulating wall ingrowth deposition in PP TCs by defoliation

**Figure 7:**
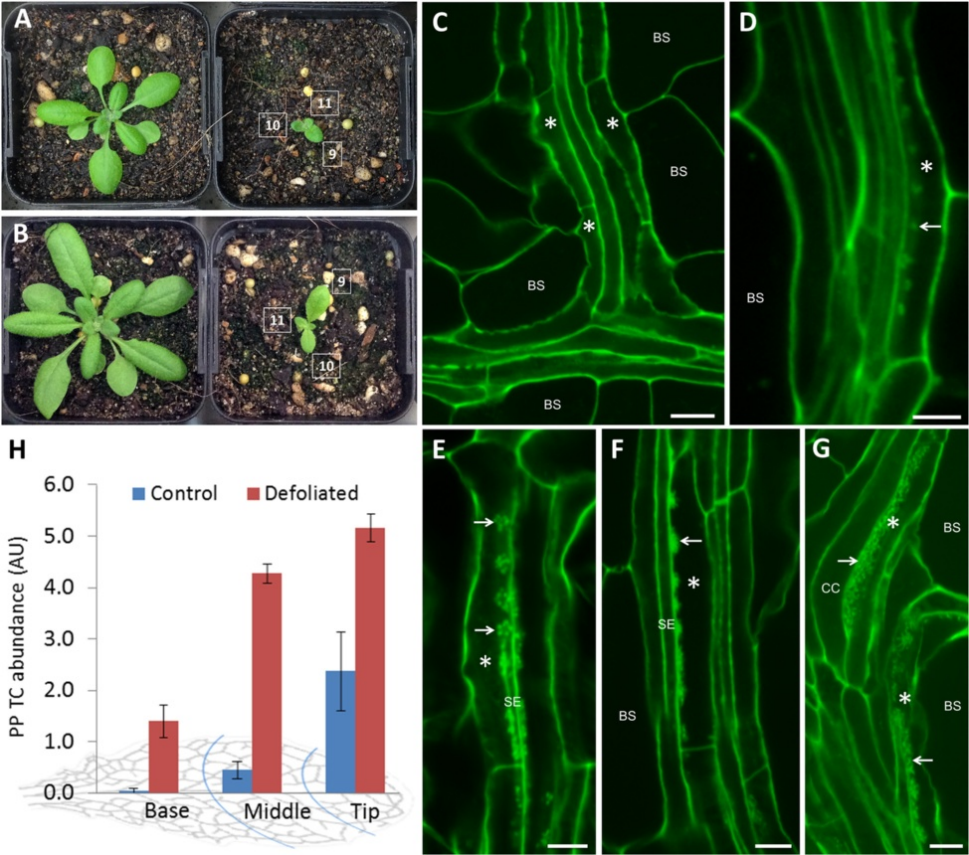
Semi-quantitative analysis of wall ingrowth deposition in PP TCs of leaf minor veins in Arabidopsis following defoliation. **A**. All leaves except leaf 9, 10, and 11 were removed from 3 week-old plants. The picture shows a control plant (left) and a defoliated plant (right) at the beginning of the experiment. **B**. Control (left) and defoliated plant (right) after 5 days additional growth. **C**. Minor vein in leaf 10 from control plant. No wall ingrowth deposition is seen in PP TCs (asterisks). **D**. Minor vein in leaf 10 from control plant showing early stage wall ingrowth deposition (arrows) in a PP TC (asterisk). **E**. Wall ingrowth deposition seen as discrete clusters (arrows) in a PP TC (asterisk) in leaf 10 of defoliated plant. **F**. Denser clusters of wall ingrowth deposition (arrow) in leaf 10 of defoliated plant. **G**. Extensive wall ingrowth deposition (arrows) in PP TCs (asterisks) in vein near the tip of leaf 10 from a defoliated plant. BS, bundle sheath; CC, companion cell. Scale bars = 5 μm in **D**, **E**, **F** and **G**. Scale bar = 10 μm in **C**. **H**. Semi-quantitative analysis of defoliation on wall ingrowth deposition in PP TCs of leaf 10 measured in control and defoliated plants 5 days after defoliation. Wall ingrowth deposition in PP TCs is greatly enhanced as a consequence of defoliation, with this response being maximal in minor veins from the middle region of the leaf. Data shows mean ± SE of scores for wall ingrowth deposition in arbitrary units (AU); *n* = 4.

We next examined the response of PP TC development to altered sink-source status in Arabidopsis leaves as a test of the mPS-PI staining procedure to provide semi-quantitative assessment of wall ingrowth development. For this analysis we conducted a defoliation experiment on 3-week-old plants by removing all leaves except leaf 9, 10 and 11 (Figure 7A). After five days further growth, leaves from control and defoliated plants (Figure 7B) were harvested and stained by the bleach-modified mPS-PI procedure. In leaf 10 from control plants, typically no wall ingrowths were visible in PP cells (Class I; Figure 7C), but in a few cases Class II wall ingrowth deposition was occasionally seen (Figure 7D). In this case, the ingrowths were small discrete clusters of wall material. In contrast to the typical lack of ingrowth deposition in control leaves, the extent of wall ingrowth deposition in leaf 10 from defoliated plants was substantially increased (Figure 7E-G). In these cases, Class III deposition was commonly seen, either in early stages of deposition where discrete clusters of tangled wall ingrowths were detected (Figure 7E), or as more dense clusters or bulges of ingrowth wall deposition (Figure 7F). In the tip region of leaf 10 from a defoliated plant, numerous cases of Class IV deposition were detected in these leaves 5 days-post defoliation (Figure 7G).

To provide a semi-quantitative analysis of the response of wall ingrowth development to defoliation, we used a scoring system whereby Class I, II, III and IV were assigned 0, 2, 4 and 6 points, respectively. Using these values, we then scored PP TC development in at least five terminating veins within the base, middle and tip regions of each leaf on either side of the mid-vein (see Figure 6B). The results of this analysis showed a rapid and significant increase in wall ingrowth deposition in PP TCs in leaf 10 of defoliated plants relative to leaf 10 in control (non-defoliated) plants, with this response being more pronounced in PP TCs in minor veins in the middle sector of leaves (ca. 8.6-fold change) compared to the tip (ca. 2.2-fold change; Figure 7H).

## Discussion

We have developed a simplified mPS-PI staining procedure for confocal imaging of wall ingrowths in vascular TCs involved in phloem loading. This procedure, involving clearing of fixed tissue using sodium hypochlorite followed by mPS-PI staining and mounting in high refractive index mounting medium provides a rapid means to image wall ingrowth deposition in TCs without the need to use more time-consuming electron microscopy techniques. The clarity of the mPS-PI staining enables high-resolution imaging of wall ingrowths in vascular tissue buried deep within leaves, and the use of sodium hypochlorite as a clearing step simplifies the original methods of Truernit et al. [16] and Wuyts et al. [17] to enable high-throughput processing of samples to suite semi-quantitative assessment of wall ingrowth deposition in TCs. Furthermore, the ability to optically section throughout an entire vascular bundle and reconstruct from a series of *z*-axis images enables the three-dimensional reconstruction of wall ingrowth deposition to analyse the highly polarised nature of this process in TCs.

We used this procedure to analyse the distribution of PP TCs both within individual leaves and in leaves of different developmental stages. Haritatos et al. [12] noted that veins of different size from mature leaves have overall similar cellular structure and organization, and since most veins are in close proximity to mesophyll, they can be considered to participate in phloem loading and thus physiologically defined as “minor” veins. Our observations support this conclusion since virtually all veins examined from mature leaves, except the midrib and larger regions of the secondary vein network, contained PP TCs with typically substantial levels of wall ingrowth deposition. Furthermore, within individual developing leaves, a basipetal gradient of PP TCs was detected (Figure 6). Both observations are consistent with development of phloem loading capacity in leaves [27,28] and demonstrate a presumed correlation between wall ingrowth deposition in PP TCs and their role in phloem loading.

The general applicability of this method for confocal imaging of wall ingrowth formation in TCs was demonstrated by visualizing CC TCs in minor veins of pea leaves. In this case, light-dependent deposition of wall ingrowths to all faces of CC TCs was clearly detected (Figure 3), consistent with earlier studies using TEM [19,20]. Thus, it is likely that this approach can be used to investigate TC development in other species and tissue locations. In Arabidopsis, we observed PP TCs with extensive wall ingrowths in sepals, cotyledons and cauline leaves (Figure 4) as well as the first true leaves of young seedlings (data not shown). Interestingly, in both cotyledons and cauline leaves, the presence of PP TCs with abundant wall ingrowths correlates with the high relative expression of both *AtSWEET11* and *AtSWEET12* (eFP Browser; bar.toronto.ca). These sucrose effluxers have recently been demonstrated to be involved in a two-step phloem loading strategy used in Arabidopsis leaves, namely unloading of sucrose into the apoplasm by PP TCs driven by AtSWEET transporters, followed by active uptake into cells of the SE/CC complex by AtSUC2 [14]. The extensive wall ingrowth deposition observed in PP TCs of cotyledons is also consistent with cotyledons acting as a strong source of photosynthesis-derived sucrose required to sustain root growth in response to light [29].

An intriguing observation seen most clearly in sepal tissue was the initial deposition of wall ingrowths as numerous discrete clusters along the length of a PP TC (Figure 4E). A similar pattern of deposition was seen in young leaves responding to defoliation (Figure 7E). These structures are presumably equivalent to the isolated patches of wall deposition observed by SEM (Figure 2D). These observations suggest that early stages of reticulate ingrowth deposition can be highly localized to discrete regions within an individual PP TC, and then continued deposition causes consolidation of these patches into more continuous regions of ingrowth deposition. The signals directing such localized patches of ingrowth deposition are unknown, but in non-vascular TC types the reactive oxygen species hydrogen peroxide has been implicated as a polarizing signal directing wall ingrowth deposition [5,30,31]. Recently, localized plumes of Ca^2+^ have been implicated in directing the highly localized deposition of individual papillae wall ingrowths in epidermal TCs of *V. faba* cotyledons [32]. A similar mechanism may operate in PP TCs, however the larger clusters of wall deposition seen by SEM and confocal imaging (Figures 2, 4 and 7) imply a higher level of organization may be operating, possibly aggregation of Ca^2+^ channels, to direct the deposition of wall ingrowths into such clusters. The ability to clearly image wall ingrowths by confocal microscopy in Arabidopsis PP TCs will enable a genetic approach to investigate signaling mechanisms driving this process.

The defoliation experiment (Figure 7) illustrates the value of the bleach-modified mPS-PI method in combination with semi-quantitative scoring to provide high-throughput and high-resolution assessment of PP TC development in leaves. The significant increase in wall ingrowth deposition in PP TCs in leaf 10 remaining after defoliation suggests a rapid switch from sink to source status within this leaf [33], and a requirement for wall ingrowth deposition for this to occur. The predicted concomitant changes in gene expression required for wall ingrowth deposition amid other processes associated with this transition provides an opportunity to identify these genes by transcriptional profiling.

## Conclusion

We have developed a simple method for confocal imaging of wall ingrowth deposition in TCs using mPS-PI staining. This method was used to image wall ingrowth deposition in PP TCs in rosette leaves, cauline leaves, cotyledons and sepals of Arabidopsis as well as CC TCs in leaf minor veins of pea. The clarity of the staining provides cellular detail of wall ingrowth deposition in these diverse tissues, thus enabling future studies investigating the cellular mechanisms directing the highly polarized deposition of wall ingrowths in TCs without the need to use electron microscopy. The high-throughput potential of this procedure also offers the opportunity to apply reverse genetics to identify genes involved in wall ingrowth deposition in TCs.

## Methods

### Plant growth conditions

*Arabidopsis thaliana* (Col-0) seeds were sown directly onto pasteurised soil mix and stratified for three days in darkness at 4°C. Plants were then transferred to a growth cabinet (100–120 μmol m^−2^ sec^−1^, 22°C day/18°C night, 16 h photoperiod) for 2–3 weeks or until stated, and cotyledons, rosette leaves, cauline leaves and sepals were then collected for analysis. Peas (*Pisum sativum*) were raised in potting mix in a glasshouse maintained at 20-24°C and approximately 800–900 μmol m^−2^ sec^−1^ during daytime. Nine days after sowing, some seedlings were covered with aluminium foil to provide dark treatment and then the second pair of true leaves from both control (full light) or dark-treated plants were harvested after 4 days further growth. For defoliation experiments, all rosette leaves except leaf 9, 10 and 11 were removed from 3-week-old Arabidopsis plants. After 5 days of additional growth, leaf 10 from both control and defoliated plants were collected and processed for mPS-PI staining.

### Pseudo-Schiff-propidium iodide staining of tissues

Rosette and cauline leaves from Arabidopsis and the second pair of true leaves from pea seedlings were pressed firmly onto clear sticky tape and the abaxial epidermal layer and associated mesophyll tissue from each leaf was peeled away using Scotch 3 M™ magic tape. Sepal and cotyledon tissue was processed without epidermal peeling. Tissue was then fixed overnight at 4°C in ethanol:acetic anhydride (3:1), then washed in 70% (v/v) ethanol and processed at room temperature as described below or stored in 70% ethanol at 4°C for several months. Tissue was washed in chloroform for 10 min, then progressively rehydrated and cleared in 1% (w/v) SDS in 0.2 N NaOH for 10 min. The extracted tissue was washed extensively in water and then incubated overnight at 37°C in 0.5% (v/v) amylase and 0.5% (v/v) pullulanase (Sigma, Australia) to remove starch. Tissue was then washed in water and incubated in 1% (v/v) periodic acid for 15 min, then washed in water again and stained in pseudo-Schiff propidium iodide for 1 h (100 mM Na_2_S_2_O_5_, 0.15 N HCl with propidium iodide added to a final concentration of 100 μg/mL at the time of staining). Stained leaves were washed briefly in water and then mounted in chloral hydrate (4 g chloral hydrate, 1 mL glycerol, and 2 mL water) with the abaxial surface of the leaf facing up. The mounted tissue was covered with a coverslip and left overnight in darkness at room temperature before viewing.

### Simplified extraction of tissue using sodium hypochlorite

Tissue was processed and fixed as described above and washed in 70% (v/v) ethanol. Cellular content of tissue was cleared by extracting tissue in 0.25% (v/v) White King™ bleach (4% (v/v) effective hypochlorite concentration) with gentle shaking at room temperature for at least 2 h depending on the tissue type. Cleared tissue was washed extensively in water and subjected to mPS-PI staining and mounting as described above.

### Confocal microscopy and image acquisition

Confocal imaging of stained tissues was performed using an Olympus FluoView FV1000 confocal microscope. Imaging used 488 nm Argon-ion laser excitation and a 60 × 1.35NA Olympus oil-immersion objective. Emission wavelengths were collected at 522–622 nm. Image pixel resolution was set at 1600 × 1600, and used pixel dwell time of 4 μs and one-way scanning. Kalman average filtering of 4 was used during image acquisitions to improve signal-to-noise ratio of the acquired images.

### Scanning electron microscopy

SEM analysis of PP TCs in mature rosette leaves was performed as described in Edwards et al. [22].

## Additional files

Additional file 1: Movie S1.
*z*-stack scan of Arabidopsis leaf minor vein showing wall ingrowth deposition in PP TCs. The scan moves through the tissue in an abaxial to adaxial direction. Wall ingrowths (red arrows) showing polarized deposition to the face of two PP TCs neighboring a common companion cell (CC) are seen. In some regions of the minor vein, wall ingrowth deposition can be seen as a central band (yellow arrows) which co-aligns with a neighboring sieve element (SE). Additional file 2: Movie S2.
*z-*stack scan of Arabidopsis second order vein showing a rare example of wall ingrowth deposition (purple arrows) in a PP TC in the vein. The scan moves through the tissue in an abaxial to adaxial direction. Most second order veins do not contain PP TCs. Wall ingrowth deposition (yellow arrows) is seen in a PP TC in the third order vein at the top of the image. M, myrosin cell; SE, sieve element. Additional file 3: Movie S3.
*z*-stack scan of Arabidopsis leaf minor vein from fixed tissue extracted with bleach. The scan moves through the tissue in an abaxial to adaxial direction. Bleach extraction provides good preservation of cellular architecture and imaging of PP TCs with imaging of wall ingrowths in PP TCs being comparable if not superior to the more complex extraction procedures employed by Truernit et al. [16] and Wuyts et al. [17]. Additional file 4: Figure S1. Additional examples of wall ingrowth deposition in PP TCs in cotyledons, cauline leaves and sepal. A, A’ and A”. Cotyledons. A. Single confocal section of a minor vein junction revealing polarized deposition of wall ingrowths (arrows). The dotted lines labelled *x-z* and *x’-z* correspond to the projection shown in A’ and A”, respectively. A’. *x-z* projection of a *z*-stack of the image shown in A revealing minor vein architecture in transverse section and the presence of highly-localized and very substantial deposition of wall ingrowth material occupying nearly half the cell volume (asterisk). A”. *x’-z* projection of a *z*-stack of the image shown in A revealing the longitudinal section of a PP TC (double asterisks) with less extensive wall ingrowth deposition but with finger-like projections (arrow). B, B’ and B”. Cauline leaves. B. Single confocal section of a minor vein junction showing polarized deposition of wall ingrowths (arrows). The dotted lines labelled *y-z* and *x-z* correspond to the projections shown in B’ and B”, respectively. B’. *x-z* projection of a *z*-stack of the image shown in B revealing minor vein architecture in transverse section and the presence of highly-localized wall ingrowth deposition (arrow). B”. *y-z* projection of a *z*-stack of the image shown in B revealing finger-like projections (arrows) of wall ingrowths in a PP TC (double asterisks). C and C’. Sepals. C. Single confocal section of a minor vein revealing polarized deposition of wall ingrowths (arrows). Note that xylem elements (XE) were also detected in this small minor vein. The dotted line labelled *y-z* corresponds to the projections shown in C’. C’. *y-z* projection of a *z*-stack of the image shown in C revealing finger-like wall ingrowth projections (arrows) in a PP TC (asterisk). Scale bars = 10 μm. Additional file 5: Figure S2. Additional examples of the four classes of wall deposition in PP TCs in Arabidopsis leaf veins. Asterisks in all figures represent PP or PP TCs. The double asterisks in I and J represent PP TCs with Class II deposition in a region of minor vein otherwise defined as Class III. Arrows point to wall ingrowth deposition in PP TCs. See Figure 5 for description of each class. Scale bars = 10 μm.

## Abbreviations

CC: Companion cell
mPS-PI: Modified pseudo-Schiff-propidium iodide
PP: Phloem parenchyma
SE: Sieve element
SEM: Scanning electron microscopy
TCs: Transfer cells
TEM: Transmission electron microscopy
