# Supplementary figures and images for: High-resolution confocal imaging of wall ingrowth deposition in plant transfer cells: Semi-quantitative analysis of phloem parenchyma transfer cell development in leaf minor veins of Arabidopsis
Source: BMC Plant Biol. 2015 Apr 23;15:109. doi: 10.1186/s12870-015-0483-8 (PMC4416241; doi:10.1186/s12870-015-0483-8)

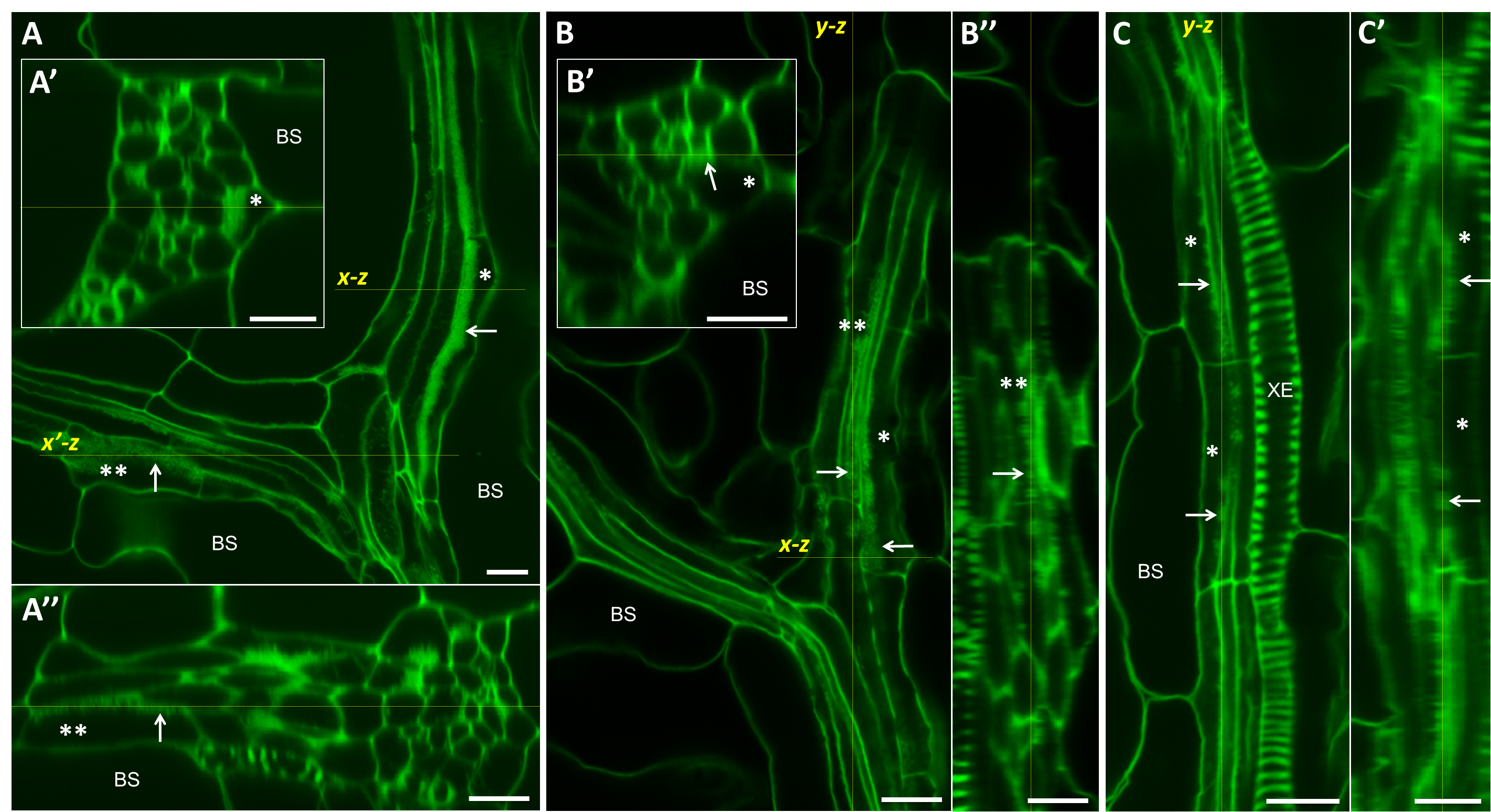

Supplement: Additional file 4: Figure S1. — Additional examples of wall ingrowth deposition in PP TCs in cotyledons, cauline leaves and sepal. A, A’ and A”. Cotyledons. A. Single confocal section of a minor vein junction revealing polarized deposition of wall ingrowths (arrows). The dotted lines labelled x-z and x’-z correspond to the projection shown in A’ and A”, respectively. A’. x-z projection of a z-stack of the image shown in A revealing minor vein architecture in transverse section and the presence of highly-localized and very substantial deposition of wall ingrowth material occupying nearly half the cell volume (asterisk). A”. x’-z projection of a z-stack of the image shown in A revealing the longitudinal section of a PP TC (double asterisks) with less extensive wall ingrowth deposition but with finger-like projections (arrow). B, B’ and B”. Cauline leaves. B. Single confocal section of a minor vein junction showing polarized deposition of wall ingrowths (arrows). The dotted lines labelled y-z and x-z correspond to the projections shown in B’ and B”, respectively. B’. x-z projection of a z-stack of the image shown in B revealing minor vein architecture in transverse section and the presence of highly-localized wall ingrowth deposition (arrow). B”. y-z projection of a z-stack of the image shown in B revealing finger-like projections (arrows) of wall ingrowths in a PP TC (double asterisks). C and C’. Sepals. C. Single confocal section of a minor vein revealing polarized deposition of wall ingrowths (arrows). Note that xylem elements (XE) were also detected in this small minor vein. The dotted line labelled y-z corresponds to the projections shown in C’. C’. y-z projection of a z-stack of the image shown in C revealing finger-like wall ingrowth projections (arrows) in a PP TC (asterisk). Scale bars = 10 μm. [file 12870_2015_483_MOESM4_ESM.tiff]

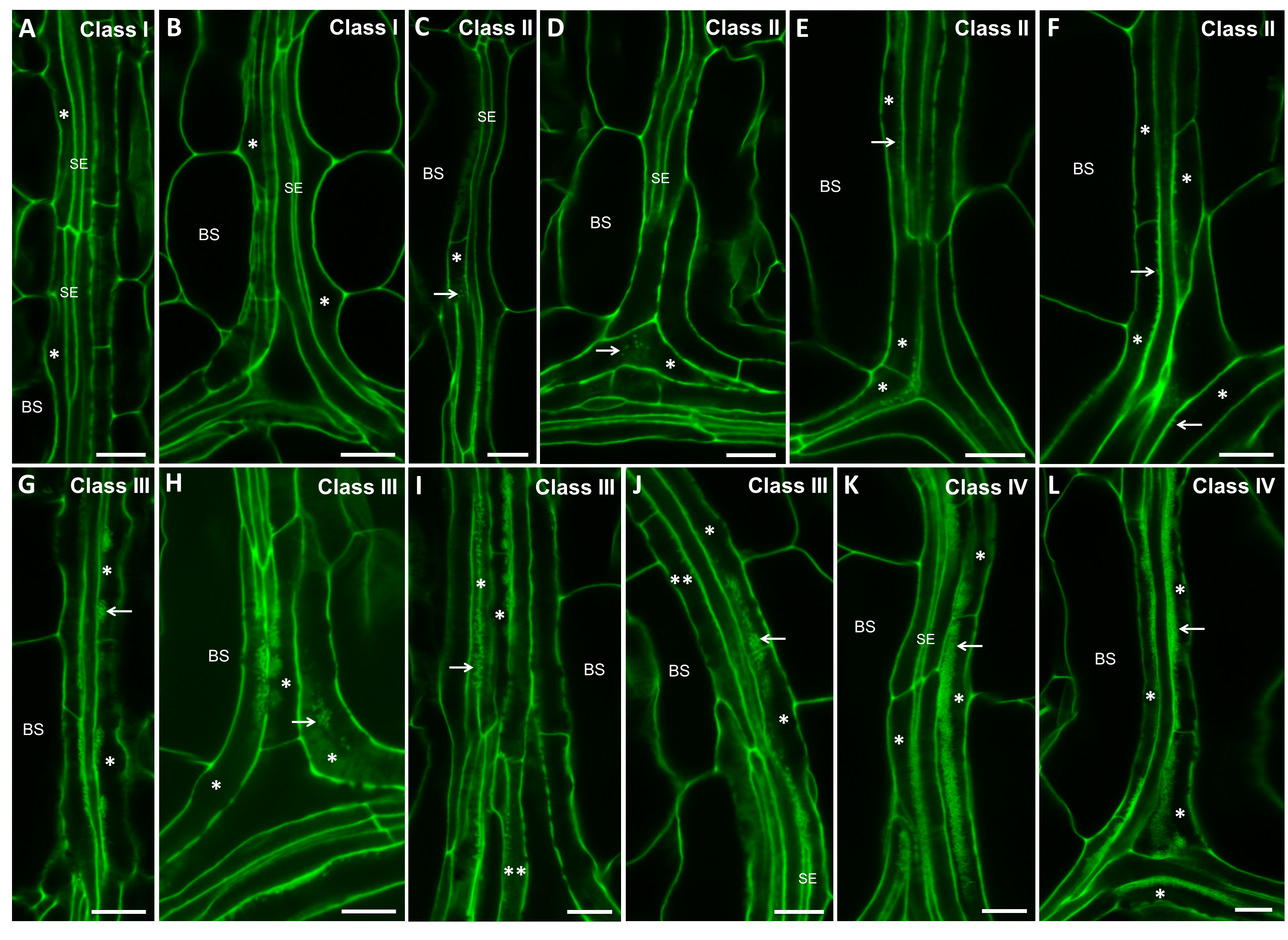

Supplement: Additional file 5: Figure S2. — Additional examples of the four classes of wall deposition in PP TCs in Arabidopsis leaf veins. Asterisks in all figures represent PP or PP TCs. The double asterisks in I and J represent PP TCs with Class II deposition in a region of minor vein otherwise defined as Class III. Arrows point to wall ingrowth deposition in PP TCs. See Figure 5 for description of each class. Scale bars = 10 μm. [file 12870_2015_483_MOESM5_ESM.tiff]
